# Supplementary material for: Iron Oxide Nanoparticles with Supramolecular Ureido-Pyrimidinone Coating for Antimicrobial Peptide Delivery
Source: Int J Mol Sci. 2023 Sep 27;24(19):14649. doi: 10.3390/ijms241914649 (PMC10573039; doi:10.3390/ijms241914649)
Supplement: Supplementary file 1 [file ijms-24-14649-s001.zip › ijms-2618690-supplementary.pdf]

Iron Oxide Nanoparticles with Supramolecular Ureido-Pyrimidinone Coating for Antimicrobial Peptide Delivery

Chiara Turrina [1], Jennifer Cookman [2], Riccardo Bellan [3], Jiankang Song [3], Margret Paar [4], Patricia Y. W. Dankers [3], Sonja Berensmeier [1] and Sebastian P. Schwaminger [1,4,5,*]

1 Chair of Bioseparation Engineering, School of Engineering and Design, Technical University of Munich, Boltzmannstr. 15, 85748 Garching, Germany; c.turrina@tum.de (C.T.)
2 Department of Chemical Sciences, Bernal Institute, University of Limerick, V94 T9PX Castletroy, Ireland; jennifer.cookman@ul.ie
3 Department of Biomedical Engineering, Institute for Complex Molecular Systems, Eindhoven University of Technology, 5600 MB Eindhoven, The Netherlands; r.bellan@tue.nl (R.B.)
4 Division of Medicinal Chemistry, Otto Loewi Research Center, Medical University of Graz, Neue Stiftingtalstraße 6, 8010 Graz, Austria
5 BioTechMed-Graz, Mozartgasse 12, 8010 Graz, Austria
* Correspondence: sebastian.schwaminger@medunigraz.at; Tel.: +43-3163-8572-125

Supporting information

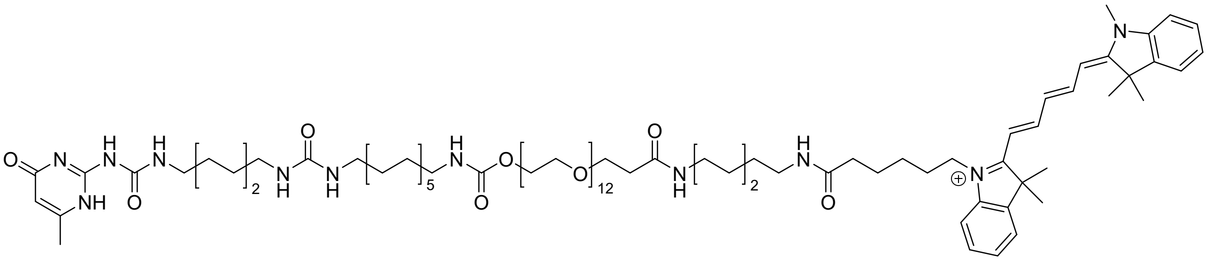

Figure S1: Structure of UPy-Cy5.

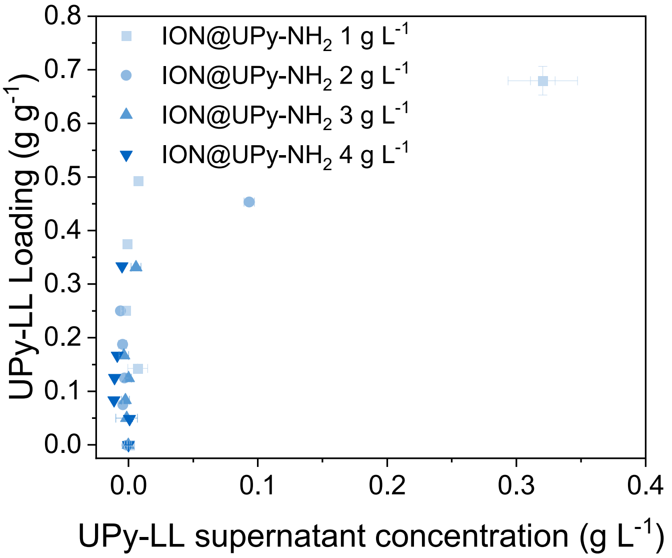

Figure S2: UPy-LL loading on different ION@UPy-NH_2 concentrations.

Table S1: Amount of bound UPy-LL on ION@UPy-NH_2 after dilution to different particle concentrations.

ION@UPy-NH_2@UPy-LL concentration [$g\ L^{-1}$]	0 $g\ L^{-1}$	0.005 $g\ L^{-1}$	0.01 $g\ L^{-1}$	0.015 $g\ L^{-1}$	0.03 $g\ L^{-1}$	0.04 $g\ L^{-1}$	0.05 $g\ L^{-1}$	0.075 $g\ L^{-1}$	0.1 $g\ L^{-1}$
UPy-LL concentration bound to ION@UPy-NH_2	0 µM	1.77 µM	3.53 µM	5.40 µM	10.6 µM	14.1 µM	17.7 µM	26.5 µM	35.3 µM

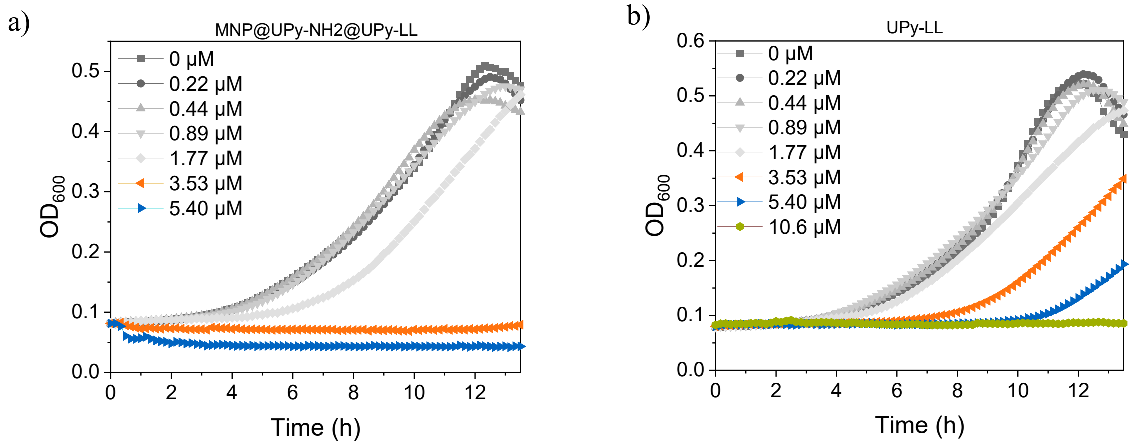

Figure S3: OD600 measurements of *E.coli* growth contacted with (**a**) ION@UPy-NH_2@UPy-LL and (**b**) free UPy-LL.
